# Feasibility of Endoscopic Closure Method Using Low Cost Clips With Thread for Post Gastric Endoscopic Submucosal Dissection: A Pilot Study

**DOI:** 10.1002/jgh3.70376

**Published:** 2026-02-27

**Authors:** Ryosuke Ikeda, Hiroaki Kaneko, Hiroki Sato, Yuto Matsuoka, Tomomi Hamaguchi, Aya Ikeda, Yoshihiro Goda, Soichiro Sue, Kuniyasu Irie, Shin Maeda

**Affiliations:** ^1^ Department of Gastroenterology Yokohama City University Graduate School of Medicine Yokohama Japan

**Keywords:** clip‐with‐thread method, cost‐effectiveness, delayed bleeding, endoscopic submucosal dissection, low‐cost endoscopic closure

## Abstract

**Background and Aim:**

Endoscopic closure in gastric endoscopic submucosal dissection (ESD) is useful to prevent delayed bleeding. Although several closure methods have been reported, their cost remains a significant issue. In this pilot study, we developed a low‐cost endoscopic closure (LoCC) method and evaluated its feasibility and cost‐effectiveness.

**Methods:**

We retrospectively analyzed 20 gastric lesions in 18 patients who underwent ESD between September 2024 and July 2025. Mucosal defect closure was performed using the LoCC method, which involves the application of conventional clips and threads to approximate mucosal edges. The primary outcome was the complete closure rate. The secondary outcomes were closure time, number of clips used and their cost, sustained closure rate on postoperative day (POD) 1, and incidence of delayed bleeding.

**Results:**

Complete closure was achieved in 90.0% (18/20) of the lesions, with sustained closure on POD1 in 85.0% (17/20). The median closure time was 17.5 min (interquartile range [IQR]: 12.3–24.0), using a median of 20 clips (IQR: 15–23), at a median cost of 131.8 United States dollars (IQR: 98.8–151.5). No cases of delayed bleeding occurred.

**Conclusions:**

The LoCC method showed favorable technical feasibility and enabled cost‐efficient closure of post‐ESD ulcers compared with other closure methods. This technique achieved a high closure success rate and sustained closure rate without the need for expensive devices, suggesting that it may serve as a practical and feasible closure method in routine clinical practice.

## 1 Introduction

Endoscopic submucosal dissection (ESD) is widely accepted as a minimally invasive treatment for early gastric cancer [1]. One of the major adverse events of gastric ESD is delayed bleeding, which has an overall incidence of 5.1% [2]. However, the delayed bleeding rate in high‐risk cases, such as patients taking antithrombotic agents or receiving hemodialysis, is approximately 11.4%–29.7% [3]. Patients taking multiple antithrombotic agents or direct oral anticoagulants (DOACs) are also at high risk of delayed bleeding [4]. Therefore, prophylactic measures are needed to prevent delayed bleeding. Several endoscopic closure techniques have recently been reported to prevent post‐ESD bleeding [5, 6, 7, 8, 9, 10, 11, 12, 13, 14, 15, 16, 17, 18]. One of the suture methods, the Reopenable Clip Over‐the‐Line Method (ROLM), reported by Nomura et al. [5], uses a reopenable clip and a thread such as nylon. Although ROLM allows for stable closure, the high cost of reopenable clips (SureClip; Micro‐Tech Endoscopy, Nanjing, China; 24 United States dollars [USD] per clip) remains an issue, as it typically requires 20–30 clips per procedure. Therefore, we devised a low‐cost endoscopic closure (LoCC) method using a conventional clip (EZ Clip; Olympus Medical Systems Co., Tokyo, Japan; 7 USD per clip) and thread and aimed to evaluate its feasibility and cost‐effectiveness in a pilot study.

## 2 Methods

### 2.1 Study Design and Patients

This single‐center, retrospective study was conducted at Yokohama City University Hospital and was approved by the Clinical Ethics Committee of Yokohama City University Graduate School of Medicine in accordance with the Declaration of Helsinki (approval No. F220600005). We included patients aged ≥ 20 years who underwent ESD for gastric adenoma or early gastric cancer between September 2024 and July 2025. We excluded patients with lesions involving the cardia or pyloric ring, which may develop stenosis due to suturing.

### 2.2 ESD Procedure

Preoperative evaluation was performed using magnifying endoscopy with narrow band imaging (GIF‐XZ1200; Olympus Medical Systems Corporation, Tokyo, Japan) to accurately confirm the tumor margins. All procedures were performed under conscious sedation with midazolam or propofol and pentazocine. ESD was performed using the conventional procedure with a single‐channel endoscope equipped with a water jet (GIF‐H290T; Olympus Medical Systems Corporation, Tokyo, Japan) and a high‐frequency power supply unit (VIO3; ERBE, Tübingen, Germany) for electrocoagulation. After marking around the tumor, a 10% glycerin solution mixed with sodium hyaluronate (MucoUp; Johnson & Johnson Medical Company, Tokyo, Japan) was injected. Mucosal incision and submucosal dissection were performed using a dual knife and an insulated‐tip knife‐2 (Olympus Medical Systems Co., Tokyo, Japan). The remaining visible blood vessels in the artificial ESD ulcer were cauterized using hemostatic forceps (Coagrasper; Olympus Medical Systems Co., Tokyo, Japan).

### 2.3 Endoscopic Closure

**FIGURE 1:**
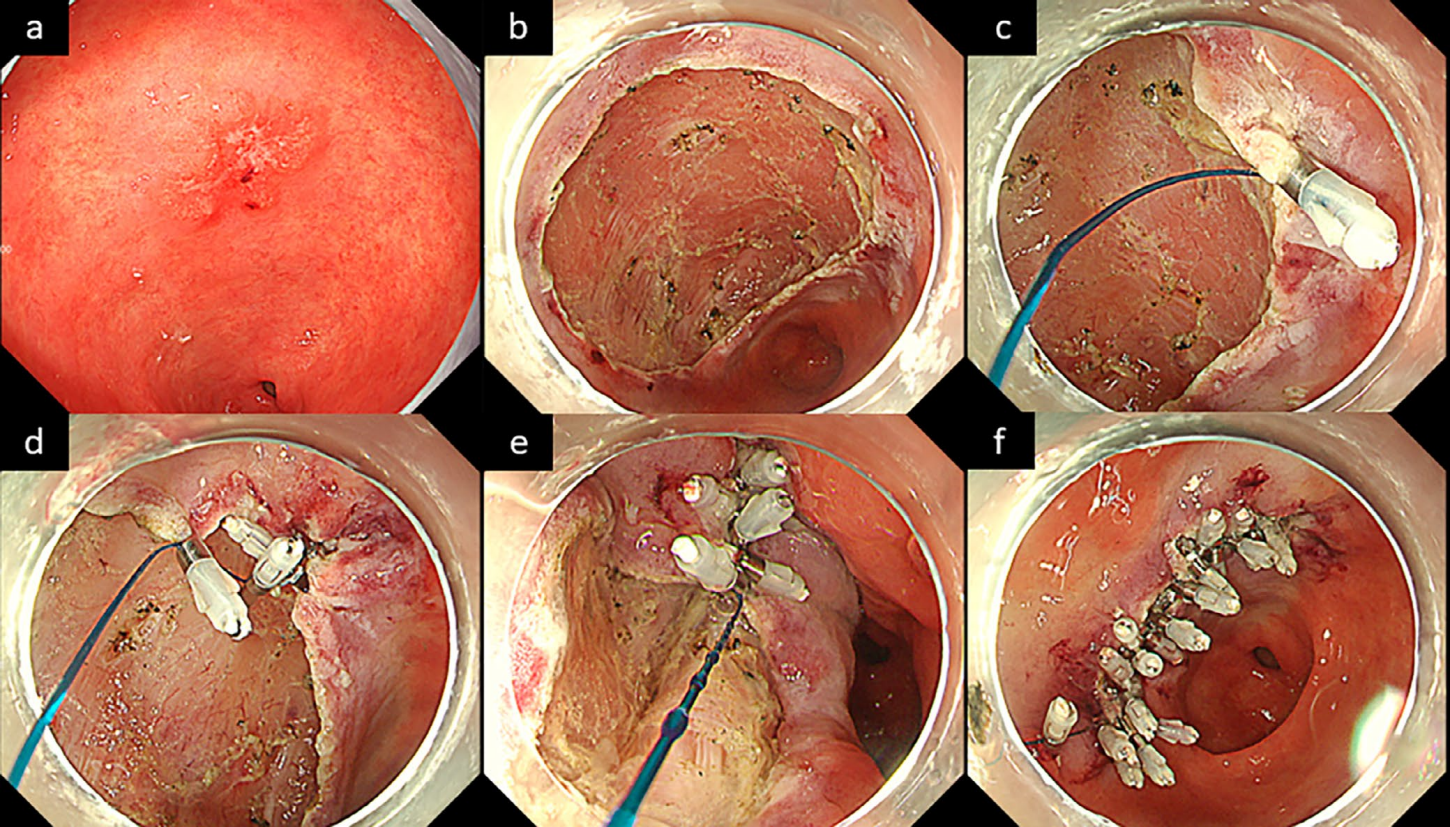
Endoscopic closure using a low‐cost clip with a thread. (a) White‐light imaging revealing a reddish superficial elevated and depressed lesion on the lesser curvature of the antrum. (b) A 43 mm post‐endoscopic submucosal dissection ulcer. (c) A clip with a thread is placed on the mucosa at the edge of the ulcer. (d) The thread is anchored to the opposite side of the mucosa using another clip. (e) Both edges of the ulcer are gathered by pulling the thread. (f) Complete closure is achieved.

Following endoscopic resection, mucosal defect closure was performed using conventional endoscopic clips (EZ Clip; Olympus) and a 3–0 nylon monofilament thread (CJ Dental Materials, Seoul, Korea) (Figure 1; Video S1). First, a thread was tied to the handle of the initial clip, which was retracted into the sheath and inserted through the working channel. The mucosa at the edge of the ESD ulcer was grasped using the initial clip with the thread. Next, a second clip was placed on the opposite side of the ulcer while grasping the thread, and the mucosal edges were approximated by pulling the thread. Subsequently, the ulcer was closed from the edge using the same procedure; however, if only the mucosa was grasped, a dead space would be created in the submucosal layer, preventing complete closure and leading to early dehiscence. Therefore, an additional clip was placed on the muscular layer of the ulcer as an anchor to ensure stable closure. This procedure eliminated the dead space in the submucosa and promoted sustained closure. After completely closing the ulcer, the thread was fixed to the mucosa with a clip and cut using a loop cutter (FS‐5 L‐1; Olympus Medical Systems Corp., Tokyo, Japan). The detailed procedural steps are shown in Video S1.

### 2.4 Management After ESD

After ESD, a second‐look endoscopy (SLE) was performed on postoperative day (POD) 1 to assess the condition of the ESD ulcers. Fluid intake was resumed 2 h after SLE, liquid meals were reinstated on POD 2, and antithrombotic agents were resumed if they had been discontinued. The patients without adverse events were discharged on POD 4. Administration of either a proton pump inhibitor or a potassium‐competitive acid blocker was initiated on POD 2 and continued for at least 4 weeks.

### 2.5 Antithrombotic Agent Management

The antithrombotic agents included in this study were the following: antiplatelet agents (aspirin, thienopyridine, and cilostazol), anticoagulants (warfarin and DOACs), and multiple antithrombotic agents (e.g., dual antiplatelet therapy or a combination of antiplatelet and anticoagulant agents). To determine drug continuation or withdrawal, we consulted the attending physician of the specialty department before planning the procedure and determined the feasibility and duration of drug withdrawal according to the Japan Gastroenterological Endoscopy Society guidelines [19, 20].

### 2.6 Outcome Measures

The primary outcome was the technical success rate of ESD ulcer closure. Successful closure was defined as complete closure of the mucosa with no endoscopically visible ulcer base. The secondary outcomes were closure time, number of clips used and their cost, sustained closure rate on POD 1, and the delayed bleeding rate. The closure time was defined as the duration from the placement of the initial clip to the cutting of the thread after the completion of closure. The cost of closure was adjusted to Japanese yen (JPY) based on 2025 values and converted to USD using the average exchange rate in 2025 (1 USD = 148 JPY). Sustained closure on POD 1 was defined as a state in which the ulcer base was not endoscopically visible and the closure remained intact without dehiscence. In addition, closure integrity beyond POD 1 was indirectly assessed using plain abdominal radiography as a non‐invasive surrogate method. Plain radiographs were obtained at 2 and 4 weeks after ESD to evaluate clip retention. Radiographic closure integrity was defined as regular alignment and retention of clips. Partial clip loss, disorganization of clip alignment, or complete clip loss was defined as loss of closure integrity (Figure S1). Delayed bleeding was defined as an episode of hematemesis or melena, or a decline in hemoglobin levels of ≥ 2 g/dL, and the requirement of emergency endoscopic hemostasis.

### 2.7 Statistical Analyses

Continuous variables are expressed as medians with interquartile ranges (IQRs), whereas categorical variables are presented as numbers and percentages. Descriptive statistics were used to summarize the patient characteristics and procedural outcomes. All statistical analyses were performed using JMP Pro version 17.0 (SAS Institute Inc., Cary, North Carolina, USA).

## 3 Results

### 3.1 Clinicopathological Features

We included 20 gastric lesions in 18 patients who underwent ESD between September 2024 and July 2025 (Table 1). The patients included 14 males and six females; the median age was 76.5 years (IQR: 71.3–81.8). Lesions were located in the upper, middle, and lower stomach in 5.0%, 20.0%, and 75.0% of the patients, respectively. Multiple synchronous lesions were observed in two patients (10.0%). The median tumor and specimen sizes were 7.0 mm (IQR: 4.0–16.5) and 32.0 mm (IQR: 24.3–36.8 mm). Two patients (10.0%) were taking antithrombotic agents, both of whom were on DOACs. One patient (5.0%) underwent hemodialysis. According to the Bleeding after ESD Trend from Japan (BEST‐J) score [3], the delayed bleeding risk was categorized as low risk in 11 lesions (55.0%), moderate in six (30.0%), high in three (15.0%), and very high in none.

**TABLE 1:** Clinicopathological features of patients and lesions.

	*N* = 20		*N* = 20
**Median age (IQR)**	76.5 (71.3–81.8)	**Depth of invasion**	
**Sex**		Mucosa, % (n)	90.0 (18)
Male, % (n)	70.0 (14)	Submucosa, % (n)	10.0 (2)
Female, % (n)	30.0 (6)	**Histopathological finding**	
**Location‐1**		Differentiated type, % (n)	95.0 (19)
Upper, % (n)	5.0 (1)	Undifferentiated type, % (n)	5.0 (1)
Middle, % (n)	20.0 (4)	**Antithrombotic agents**	
Lower, % (n)	75.0 (15)	(+), % (n)	10.0 (2)
**Location‐2**		(−), % (n)	90.0 (18)
Anterior wall, % (n)	20.0 (4)	**Hemodialysis**	
Posterior wall, % (n)	30.0 (6)	(+), % (n)	5.0 (1)
Lesser curvature, % (n)	45.0 (9)	(−), % (n)	95.0 (19)
Greater curvature, % (n)	5.0 (1)	**BEST‐J score (IQR)**	1.0 (0.3–2.0)
**Morphology**		**BEST‐J score risk**	
Protruded, % (n)	40.0 (8)	Low, % (n)	55.0 (11)
Flat/depressed, % (n)	60.0 (12)	Middle, % (n)	30.0 (6)
**Synchronous occurrence, % (n)**	10.0 (2)	High, % (n)	15.0 (3)
**Median tumor size (IQR)**	7.0 (4.0–16.5)	Very high, % (n)	0 (0)
**Median specimen size (IQR)**	32.0 (24.3–36.8)	**BEST‐J score (IQR)**	1.0 (0.3–2.0)
**Ulcerative findings**			
(+), % (n)	10.0 (2)		
(−), % (n)	90.0 (18)		

Abbreviations: BEST‐J, bleeding after endoscopic submucosal dissection trend from Japan; IQR, interquartile range.

### 3.2 ESD Treatment Outcomes

The en bloc, R0, and curative resection rates were all 100% (Table 2). No intra‐operative or delayed perforations were observed. The median procedure time was 14.5 min (IQR: 10.0–27.5).

**TABLE 2:** ESD treatment outcomes.

		*N* = 20
En‐bloc resection, % (*n*)		100 (20)
R0 resection, % (*n*)		100 (20)
Curative resection, % (*n*)		100 (20)
Median procedure time (IQR)		14.5 (10.0–27.5)

Abbreviations: ESD, endoscopic submucosal dissection; IQR, interquartile range.

### 3.3 LoCC Method Outcomes

The primary and secondary outcomes are shown in Table 3. The complete closure rate was 90.0% (18/20 lesions). As a secondary outcome, the median closure time was 17.5 min (IQR: 12.3–24.0). The median number of clips used per lesion was 20 (IQR: 15–23), corresponding to a total cost of 131.8 USD (IQR: 98.8–151.5). The sustained closure rate on POD 1 was 85.0% (17/20). Radiographic closure integrity, assessed as an indirect surrogate beyond POD 1, was maintained in 55.0% (11/20) of lesions at 2 weeks and 25.0% (5/20) at 4 weeks. No cases of delayed bleeding were reported.

**TABLE 3:** Primary and secondary outcomes.

	*N* = 20
Primary outcome	
Complete closure rate, % (n)	90.0 (18)
Secondary outcome	
Sustained closure rate on POD 1, % (n)	85.0 (17)
Procedure time of closure (IQR)	17.5 (12.3–24.0)
Number of clips (IQR)	20 (15–23)
Cost of closure, USD (IQR)	131.8 (98.8–151.5)
Delayed bleeding, % (n)	0 (0)

Abbreviations: IQR, interquartile range; POD, postoperative day; USD, United States dollars.

## 4 Discussion

Delayed bleeding in gastric ESD remains a major concern, particularly in patients categorized as high‐or very high‐risk according to the BEST‐J score [3]. With an aging population and an increasing prevalence of cardiovascular and cerebrovascular diseases, the number of patients receiving antithrombotic therapy is increasing. Therefore, endoscopic closure to prevent delayed bleeding in these patients is important.

Several endoscopic closure methods have recently been reported. The closure method using clips alone is technically simple and cost‐effective; however, its success rate is limited to 62%, and incomplete closure increases with larger mucosal defects [6]. Closure methods using clips and endoloops [9, 10] enable closure even for large ulcers, provided the defect does not exceed the loop size. These methods showed a favorable closure success rate (86.3%–89.0%). However, dead space may develop beneath the sutured mucosa due to the purse‐string suture, resulting in a low sustained closure rate of 47.8% on POD 1 [10]. Moreover, loop‐assisted closure is technically challenging in anatomically angulated locations, and the procedure requires a certain degree of technical proficiency. Endoscopic hand suturing (EHS) [13] and the over‐the‐scope clip (OTSC) system (Ovesco Endoscopy AG, Tübingen, Germany) [14] achieved a significant closure strength. EHS enables secure mucosal closure by directly suturing the mucosa with a curved suture needle, whereas OTSC uses a dedicated device that allows for robust closure by grasping the tissue, including the muscularis propria. These methods showed high closure success rates (100% for EHS and 91.7% for OTSC). However, both devices are expensive (EHS: 831 USD, OTSC: 539 USD), and EHS requires advanced endoscopic suturing skills. ROLM is performed by passing a thread through the hole of the SureClip and placing the clip at the edge of the mucosal defect to approximate the tissue by pulling the thread. This technique has been reported to achieve complete closure even in large ESD ulcers with a high closure success rate (100%), making it a valuable closure method. As the mucosal defect gradually closes from the edges, dead space formation is minimized, which is expected to contribute to favorable sustained closure [21]. However, SureClip is relatively expensive (24 USD per clip), and the total cost increases substantially when multiple clips are required. Therefore, we developed a novel closure method using an inexpensive clip to achieve cost‐effectiveness and favorable complete closure rates. Closure techniques using clips and threads have been reported previously [15, 16, 17, 18]. However, these methods use thread‐assisted clips to approximate a partial area of the defect, whereas the remaining defect is closed using clips only. The LoCC method achieves complete closure by sequentially approximating the mucosa of the defect using low‐cost clips. We investigated the feasibility of the LoCC method in this pilot study.

The LoCC method showed a favorable closure success rate of 90.0%, with a sustained closure rate of 85.0% on POD 1. These outcomes are comparable to those of other closure methods that use more expensive devices and endoloop‐assisted closure methods. In this study, radiographic clip retention was additionally evaluated at 2 and 4 weeks after ESD as an indirect assessment of closure integrity beyond POD 1. Although direct endoscopic confirmation of the ESD ulcer would be the most reliable method for evaluating sustained closure, repeated endoscopic examinations would be required and may increase patient burden. Therefore, we assessed closure integrity using plain abdominal radiographs as a non‐invasive surrogate method. At 2 weeks, regular clip alignment was observed in 55% of cases. These findings suggest that closure integrity may be maintained in approximately half of the cases during the early post‐procedural period of around 2 weeks. Moreover, the median procedure time for closure was 17.5 min, which is clinically acceptable compared with previously reported methods. Among other methods, endoloop‐assisted closure methods have been reported to require 15–33 min; EHS approximately 48 min, and OTSC closure 15.1 min. Therefore, the LoCC method has the potential to be a closure technique that can be performed without excessively prolonging procedure times. Delayed bleeding did not occur in this study, suggesting that the LoCC method may prevent delayed bleeding. However, this pilot study was designed to evaluate the technical feasibility of the LoCC method; further accumulation of patients, particularly those at high to very high risk, is required to evaluate the efficacy of the LoCC method in preventing delayed bleeding.

The LoCC method presents several technical challenges. While the ROLM technique uses reopenable clips that allow repositioning and regrasping of the thread or mucosa, the LoCC method uses non‐reopenable clips, making precise initial placement difficult. In particular, the procedure may be technically challenging at anatomical locations where a retroflexed view is required. In our study, there were two cases of unsuccessful closure. At the gastric angle, the closure was performed under a retroflexed view, which may have resulted in poor maneuverability. However, all subsequent gastric angle cases were successfully closed, suggesting that proficiency in the technique improved with practice. In another case, at the anterior wall of the upper gastric body, the antegrade endoscopic view was unstable, and insufflation was required to maintain an adequate working space. Consequently, it was difficult to approximate the mucosa under air deflation. Because sufficient working space is also necessary to grasp the thread with a clip, the LoCC method under air‐deflated or underwater conditions may be more difficult than ROLM.

Nevertheless, a notable advantage of the LoCC method is its cost‐effectiveness (Table S1). Although other closure methods, such as EHS, OTSC, and ROLM, have shown high closure success rates, the closure cost of each clip or device is considerably high (EHS: 831 USD, OTSC: 539 USD, and ROLM: 24 USD), and ROLM typically requires 20–30 clips per procedure, resulting in a total closure cost of approximately 480–720 USD. In cases with large mucosal defects requiring multiple devices, the total cost per procedure can increase substantially compared to the LoCC method. In contrast, the EZ Clip used in this study was priced at approximately 7 USD per clip, and the total cost remained at approximately 130 USD, even with a median usage of 20 clips per case. This is approximately one‐third of the cost of SureClips (24 USD per clip). If 20 SureClips are used in ROLM, the total cost reaches approximately 480 USD. Thus, the LoCC method achieves a high closure success rate and sustained closure without the need for expensive dedicated devices, and it is considered a feasible and cost‐effective method within an insurance‐based healthcare system.

This study had several limitations. First, it was a retrospective analysis conducted at a single center with a relatively small sample size, because this was a pilot study. Second, the lesions included in this study had a median resection diameter of approximately 30 mm; therefore, the efficacy and safety of the LoCC method for larger mucosal defects require further investigation. Third, the sustained closure rate beyond POD 1 was not directly evaluated using endoscopy. Although radiographic clip retention was used as an indirect surrogate of closure integrity, it does not necessarily reflect complete mucosal closure; therefore, a more detailed investigation of closure sustainability would require periodic endoscopic follow‐up.

In conclusion, the LoCC method showed favorable feasibility and offered a significant cost advantage, suggesting that it may serve as an effective closure technique following gastric ESD.

## Funding

The authors have nothing to report.

## Ethics Statement

The Clinical Ethics Committee of Yokohama City University Graduate School of Medicine approved this study (approval number: F220600005).

## Conflicts of Interest

The authors declare no conflicts of interest.

## Supporting information


**Figure S1:** jgh370376‐sup‐0001‐FigureS1.tif. **Radiographic assessment of clip retention following the LoCC method**.
(a) Complete clip retention with regular alignment, defined as maintained closure integrity.
(b) Partial disorganization of clip alignment, defined as loss of closure integrity.
(c) Complete loss of clips, defined as loss of closure integrity.

**Table S1:** Cost comparison of closure methods (LoCC, EHS, OTSC, and ROLM).

**Video S1:** jgh370376‐sup‐0003‐VideoS1.mp4. **Endoscopic closure procedure using the LoCC method**.

## Data Availability

The data that support the findings of this study are available on request from the corresponding author. The data are not publicly available due to privacy or ethical restrictions.
