# Supplementary figures and images for: Feasibility of Endoscopic Closure Method Using Low Cost Clips With Thread for Post Gastric Endoscopic Submucosal Dissection: A Pilot Study
Source: JGH Open. 2026 Feb 27;10(3):e70376. doi: 10.1002/jgh3.70376 (PMC12949335; doi:10.1002/jgh3.70376)

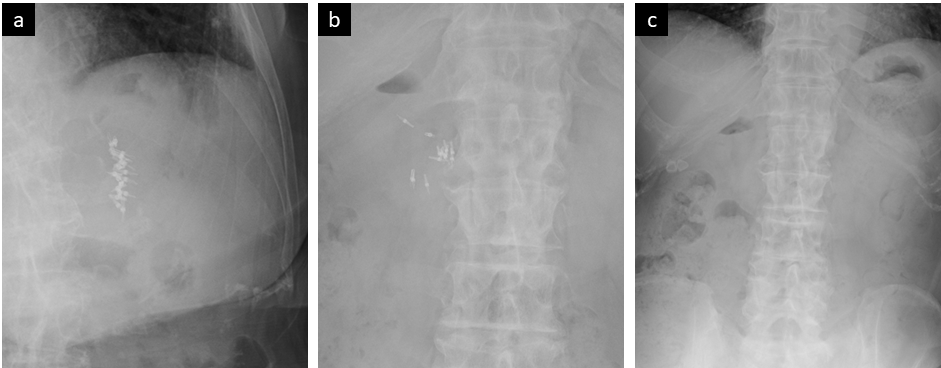

Supplement: Supplementary file 1 — Figure S1: jgh370376‐sup‐0001‐FigureS1.tif. Radiographic assessment of clip retention following the LoCC method. (a) Complete clip retention with regular alignment, defined as maintained closure integrity. (b) Partial disorganization of clip alignment, defined as loss of closure integrity. (c) Complete loss of clips, defined as loss of closure integrity. [file JGH3-10-e70376-s003.tif]
