# Supplementary material for: Feasibility of Endoscopic Closure Method Using Low Cost Clips With Thread for Post Gastric Endoscopic Submucosal Dissection: A Pilot Study
Source: JGH Open. 2026 Feb 27;10(3):e70376. doi: 10.1002/jgh3.70376 (PMC12949335; doi:10.1002/jgh3.70376)
Supplement: Supplementary file 2 — Table S1: Cost comparison of closure methods (LoCC, EHS, OTSC, and ROLM). [file JGH3-10-e70376-s001.docx]

| **Table S1. Cost comparison of closure methods (LoCC, EHS, OTSC, and ROLM)** | | | | |  |
| --- | --- | --- | --- | --- | --- |
|  |  |  |  |  |  |
| **Closure method** | **Device** | **Unit price (USD)** | **Required quantity** | **Total cost (USD)** | **Remarks** |
| LoCC | EZ Clip | 7 | Approx. 20 clips | Approx. 130 | High closure success, most cost-effective |
| ROLM | SureClip | 24 | Approx. 20 clips | Approx. 480 | High closure success, relatively expensive |
| EHS | Endoscopic hand suture device | 831 | 1 set | 831 | Robust closure, expensive |
| OTSC | OTSC system | 539 | 1 set | 539 | Robust closure, expensive |
|  |  |  |  |  |  |
| LoCC, low-cost clip closure; ROLM, reopenable clip over-the-line method; EHS, endoscopic hand-suturing; OTSC: over-the-scope clip; USD, United States dollar. | | | | | |
